# A comparison between the Felix™ electrophoretic system of sperm isolation and conventional density gradient centrifugation: a multicentre analysis

**DOI:** 10.1007/s10815-022-02680-0

**Published:** 2022-12-14

**Authors:** Farnaz Shapouri, Tara Mahendran, Mirudhubashini Govindarajan, Philip Xie, Olena Kocur, Gianpiero D. Palermo, Hassan W. Bakos, Aisling Ahlström, Gunilla Caisander, Bo Xu, Shun Bai, Sarah Lambourne, R. John Aitken

**Affiliations:** 1 Memphasys Ltd, 30 Richmond Road, Homebush West, NSW 2140 Australia; 2 Andrology Center, Coimbatore, India; 3 Womens Center and Hospitals, Coimbatore, India; 4 The Ronald O. Perelman and Claudia Cohen Center for Reproductive Medicine, Weill Cornell Medicine, New York, NY USA; 5 Monash IVF Group Limited, Level 2, 1 Fennell Street, Parramatta, NSW 2151 Australia; 6 GeneraLife IVF, 40229 Gothenburg, Sweden; 7 Reproductive and Genetic Hospital, The First Affiliated Hospital of USTC, Division of Life Sciences and Medicine, University of Science and Technology of China, Hefei, Anhui 230001 People’s Republic of China; 8 Priority Research Centre for Reproductive Science, Discipline of Biological Sciences, School of Environmental and Life Sciences, College of Engineering Science and Environment, University of Newcastle, Callaghan, NSW 2308 Australia; 9 Hunter Medical Research Institute, New Lambton Heights, NSW 2305 Australia

**Keywords:** Sperm selection, Density gradient centrifugation, Electrophoretic separation, Motility, DNA damage

## Abstract

**Purpose:**

Developing optimized techniques for the isolation of human spermatozoa possessing low levels of DNA damage is an important objective for the ART industry. The purpose of this study was to compare a novel electrophoretic system (Felix™) of sperm isolation with a conventional method involving density gradient centrifugation (DGC).

**Methods:**

Five international ART Centres in Australia, India, Sweden, the USA, and China have collaborated in order to compare the quality of the sperm populations isolated by Felix™ and DGC in terms of processing time, sperm concentration, motility, vitality, and DNA integrity as assessed by 3 methods: SCSA, Halo, and TUNEL.

**Results:**

Across all centers, 112 comparisons were performed. Although significant differences were noted between centers in terms of the quality of the semen samples subjected for analysis, overall, both methods were equally capable of isolating populations of spermatozoa exhibiting high levels of vitality and progressive motility. The absolute numbers of spermatozoa recovered were significantly (*p* < 0.001) lower with the Felix™ device although sperm quality was higher with 4/5 centers reporting a significant improvement in DNA integrity relative to DGC (*p* < 0.01–*p* < 0.001). In practical terms, the Felix™ device featured a standardized 6 min preparation time whereas clinical DGC protocols varied from center to center but generally took around 40 min to complete.

**Conclusions:**

The Felix™ device is a positive technical development capable of isolating suspensions of highly motile spermatozoa exhibiting low levels of DNA damage in a fraction of the time taken by conventional procedures such as DGC.

## Introduction

Developing optimized technologies for the preparation of spermatozoa for assisted conception is a priority for the ART industry. Essentially, the methods that are currently routinely used to prepare spermatozoa in this context have remained unchanged for more than half a century, despite the introduction of several microfluidics systems that have not, as yet, gained widespread support [1]. The most common technique in clinical practice, density gradient centrifugation, is based on the principle that the highest-quality spermatozoa in an ejaculate possess the highest isopycnic density [2]. While continuous density gradients were used historically to separate functional spermatozoa for assisted conception purposes [3], this approach was rapidly succeeded by the use of discontinuous density gradients employing multiple layers at first [4, 5] but ultimately being simplified to a 2-step [2, 6] or, occasionally, a one-step system [7]. Recovery of spermatozoa from the high-density regions of such gradients yields populations that are characterized by high levels of progressive motility, good morphology, high levels of chromatin condensation, low levels of both leukocyte and bacterial contamination, low levels of oxidative stress, and high levels of fertilizing potential [8–10]. Clinically, such density gradient techniques are as effective as swim-up in terms of the generation of clinical pregnancies [11] and may be even more effective when adapted for use with oligoasthenozoospermic samples, when both sperm number and motility may be severely compromised [12].

Notwithstanding the apparent efficacy of discontinuous gradients for the preparation of human spermatozoa in an IVF context, this methodology is associated with some inherent drawbacks. First of all, it involves the repeated centrifugation of human spermatozoa at relatively high *g* forces for prolonged periods of time. According to the WHO laboratory manual [13], the preparation of spermatozoa using a density gradient approach should involve an initial separation step during which these cells are subjected to 300–400 g for 15–30 min followed by a further two washing steps, involving additional repeated centrifugation at 200 g for 4–20 min to remove all traces of the gradient material. For some particularly vulnerable sperm samples, prolonged exposure of spermatozoa to the shearing forces associated with centrifugation appears to have a negative impact on sperm function and membrane integrity impairing motility, mitochondrial energy production, and, critically, DNA integrity [4, 14–17]. Some of this damage appears to be induced by the induction of reactive oxygen species (ROS) generation during the centrifugation procedure [4, 18, 19]. The negative impact of ROS under these circumstances may be further exacerbated by the presence of transitional metals in commercial sperm preparation media that catalyze lipid peroxidation cascades in these cells, and precipitate significant levels of oxidative DNA damage [17, 20].

In order to avoid such issues with density gradient centrifugation, a variety of alternative sperm isolation techniques have been developed that are designed to reduce the amount of stress suffered by these cells during their separation from seminal plasma. One such technique is an electrophoretic method that isolates high-quality spermatozoa on the basis of their size, motility and net negative charge [21]. A prototype electrophoretic sperm isolation device has been developed and subjected to preliminary clinical trials, where it was shown to support fertilization and normal embryo development resulting in the delivery of normal term offspring when deployed in a clinical IVF/ICSI setting [22, 23]. On the basis of these promising results, a commercial electrophoretic sperm isolation device (the Felix™ system) has been developed and is now being evaluated in extensive multicentered clinical trials. The purpose of this publication is to present the first results of a comparative analysis of this Felix™ system in comparison with DGC for the rapid isolation of high-quality human spermatozoa possessing a low level of DNA damage suitable for use in assisted conception programs.

## Materials and methods

This study is approved by the institutional ethical boards of each respective institution and informed consent was obtained from each couple. The centers involved in this study comprised the following: (1) Andrology Center, Coimbatore, India, and Women’s Center and Hospitals, Coimbatore, India; (2) The Ronald O. Perelman and Claudia Cohen Center for Reproductive Medicine, Weill Cornell Medicine, New York, USA; (3) Monash IVF Group, Sydney; (4) Livio Gothenburg, Gothenburg, Sweden; and (5) University of Science and Technology of China, China. The study received ethical approval from the respective institutional human ethics review boards (Australia, H-2013–0319; Sweden, 2019–05695; USA, IRB# 0712009553 and IRB# 1006011085; India, 22.11.20; China, 2022-RE-261).

### Semen analysis

Semen samples were produced by masturbation into a sterile container following a recommended period of sexual abstinence of at least 3 days. All patient samples were produced in a private room available within each ART laboratory site, with the exception of the Livio Gothenburg Swedish clinic, where patients were offered the possibility of producing a sample at home and then delivering it to the laboratory within 1 h of ejaculation. Semen samples were allowed to liquefy at room temperature (Australia and Sweden) or 37 °C (USA, China, and India) before being subjected to a routine semen analysis including volume, concentration, vitality, motility, and DNA fragmentation. The methodologies employed for creating the conventional semen profile varied between clinics in accordance with their standard operating procedures. Although the participating centers did not share a common external validation method, the quality management system at each clinic included an external validation scheme for sperm assessment. In all centers, non-progressive motility, progressive motility, and percentage of immotile cells were classified in accordance with the World Health Organization criteria for examination and processing of human semen (13). Sperm concentration was determined using a Makler chamber, Burker chamber, or a computer-assisted sperm analysis (CASA) system in different locations (Table 1). For vitality assessment, samples were stained with eosin and nigrosin, before a slide smear was made and vital spermatozoa were counted under oil immersion (× 100 magnification). The DNA integrity of spermatozoa was assessed using a sperm chromatin dispersion assay (Halo), a Terminal dUTP Nick-End Labeling (TUNEL) assay, or a sperm chromatin structure assay (SCSA) in individual centers as set out in Table 1 and described in detail below. Following the initial semen analysis, each sample was split between the isolation methods, density gradient centrifugation (DGC), and electrophoretic isolation (Felix™, Memphasys, Sydney, Australia) for comparative processing; 1 mL of sample was allocated to each isolation method. Whenever possible, these assessments were conducted blind; however, there were situations such as the real-time scoring of sperm movement, when this was not possible.

**Table 1:** Experimental procedures used in this study by each study center

Structure of the DGC protocols		Monash IVF (Australia)	Weil Cornell (USA)	Livio Gotenburg (Sweden)	USTC Hospital (China)	Women’s Center Hospital (India)
Number of layers		2	1	2	2	2
Name and volumes of material used to make the gradient (mL)		1 mL-SpermGrad (Vitrolife, Sweden)	1 mL-Enhance-S Plus Cell Isolation (Vitrolife, Sweden)	2 mL-Pure Sperm Wash media (Nidacon, Sweden)	2 mL-SAGE solution (In-Vitro Fertilization Inc., Trumbull, CT, USA)	1 mL-SpermGrad (Vitrolife, Sweden)
Density of each gradient layers (%)		45 over 90%	90%	40 over 90%	40 over 80%	45 over 90%
Centrifugation speed (*g*) and time (min)		300 g centrifugation for 20 min	300 g centrifugation for 10 min	300 g centrifugation for 20 min	400 g centrifugation for 20 min	290 g centrifugation for 20 min
Pellet resuspending media		2 mL G-IVF + (Vitrolife, Sweden)	*H-HTF (FujiFilm:Irvine Scientific, CA, USA) supplemented with **HSA solution (G Series culture media; Vitrolife, Sweden)	5 mL Pure Sperm Wash Media (Nidacon, Sweden)	5 mL of Spermrinse (Vitrolife, Sweden)	5 mL of Spermrinse (Vitrolife, Sweden)
Subsequent wash speed (*g*) and time (min)		300 g centrifugation for 10 min	600 g centrifugation for 10 min	500 g centrifugation for 10 min	300 g centrifugation for 6 min	290 g centrifugation for 10 min
Semen analysis methods	Concentration/motility/progr. motility	Makler chamber	Makler chamber	Burker chamber	CASA	Makler chamber
	DNA	Halo	TUNEL	Halo	Halo	SCSA
	Vitality	Eosin-Nigrosin staining	NA	NA	CASA	Eosin-Nigrosin staining

^*^HEPES-buffered human tubal fluid medium

^**^Human serum albumin

### Sperm preparation by density gradient centrifugation

The density gradient centrifugation (DGC) procedure was carried out at each site according to the individual clinic’s standard operating procedure as described in Table 1. Most clinics (4 out of 5) used a simple 2-step DGC protocol although Weil Cornell employed a single step procedure. Briefly, 1 mL of semen sample was layered over the sperm separation medium and centrifuged at 290–400 g for 10–20 min, depending on the center. Subsequently, the sperm pellet was washed with fresh medium and centrifuged again at 290–600 g (Table 1). The supernatant was then discarded and the final sample resuspended in a defined incubation medium for further analysis as set out in Table 1.

### Sperm preparation by Felix™

Sperm preparation using the Felix™ device was performed by dispensing 4 mL of G-Rinse™ medium (Vitrolife, Sweden) into each buffer chamber, followed by pipetting 1 mL G-IVF™ PLUS medium (Vitrolife, Sweden) into the harvest chamber. One milliliter of the liquefied semen sample was loaded into the sample chamber. After completion of the 6 min Felix™ system processing cycle, 0.3 mL of the isolated sperm suspension was gently removed from the harvest chamber using a glass pipette comprising a 2-mL syringe (Becton Dickinson, Sigma-Aldrich) connected to a glass pipette (20 × 5.77 inch IVF Pasteur Pipette, Origio, Cooper Surgical, Denmark) with a 2-cm silicon plastic connector (6 mm internal diameter). The extracted harvest sample was then ready further analysis.

### Sperm DNA integrity

Each IVF clinic nominated one assay that would be used to assess the DNA integrity of each sperm sample before and after the different isolation methods. These assays were the sperm chromatin structure assay (SCSA) (India and China), the sperm chromatin dispersion test (Halo assay; Australia and Sweden), and the Terminal Uridine Deoxynucleotidyl Transferase-mediated Nick-end Labeling technique (TUNEL) (USA). For the SCSA and Halo assays, 50–100 µL of each original and isolated sample was snap-frozen by immersion liquid nitrogen at − 196 °C and stored in this medium. Samples generated at the Swedish Centre that were destined for analysis using the Halo assay in Australia were shipped on dry ice with a temperature data logger (at − 78 °C) and then stored at − 80 °C prior to analysis. For the TUNEL assay, slides were smeared with 5 μL of the semen sample from before and after each selection method and left to dry overnight.

### Sperm chromatin dispersion assay

In 3 centers, levels of DNA fragmentation within each sperm sample were determined according to the sperm chromatin dispersion test or Halo assay, using the Halosperm® G2 kit (HT-HSG2, Halotech, Denmark) and the scoring protocol suggested by the manufacturers. According to this methodology, spermatozoa with fragmented DNA have no halo, a small halo (the halo width is similar or smaller than 1/3 of the diameter of the core), or no halo with a degraded, poorly staining nucleus. Samples were submitted for analysis by two centers (Australia and Sweden) and to minimize variation, the assay was performed on a single site (University of Newcastle) by a research associate blinded to the identity of individual samples. In terms of methodology, snap-frozen semen and isolated spermatozoa were thawed at room temperature before being immersed in an agarose matrix on a slide, treated with an acid solution to denature the DNA and then treated with lysis buffer to remove membranes and proteins. The cells were then stained, washed, and dried with ethanol before being analyzed under bright field microscopy. The cells were classified into 5 categories: large halo, medium halo, small halo, no halo, or degraded spermatozoa. The percentage of DNA damaged spermatozoa was given by the percentage of cells falling into the small halo, no halo, and degraded categories. For the Livio Gotenborg site, the Halo assay was performed on 50 µL aliquots of semen and spermatozoa that had been snap frozen and shipped to the Priority Research Centre for Reproductive Science at the University of Newcastle, Australia.

### The sperm chromatin structure assay

The SCSA method measures the susceptibility of sperm DNA to acid-induced DNA denaturation in situ, followed by staining with the fluorescent dye acridine orange [24]. For this study, a flow cytometer was used to analyze the cells. Snap frozen raw semen and isolated sperm samples stored in liquid nitrogen tanks were thawed in a water bath and diluted with TNE buffer (0.01 M Tris–HCl: 0.15 M NaCl: 1 mM EDTA, pH 7.4, Sigma-Aldrich). The sperm suspension was mixed with an acid solution containing: 0.08 M HCl (Titripur, Sigma-Aldrich), 0.15 M NaCl (Sigma-Aldrich) 0.1% (v:v) Triton X-100 (Sigma-Aldrich) at 4 °C for DNA denaturation. After 30 s, the spermatozoa were stained with a staining solution containing 6 µg/mL acridine orange (AO, chromatographically purified, Polysciences, Inc., Warrington, PA, USA), 0.2 M Na_2_PO_4_: 0.1 M Citric acid (pH 6.0): 1 mM EDTA: 0.15 M NaCl (Sigma-Aldrich). The stained sample was run on a FACSCalibur™ flow cytometer (BD Biosciences, San Jose, CA, USA) and 5000 spermatozoa were analyzed at an event rate of 100–250 events/s. The flow cytometer was calibrated with a reference sample at the start of sample analysis, and the same reference sample was analyzed after every five test samples to calibrate the instrument. Each sample was analyzed in duplicate, and replicates of the data were utilized to determine the percentage of spermatozoa with measurably increased red fluorescence (the DNA fragmentation index [DFI] as determined using proprietary software (SCSA soft®, SCSA Diagnostics, Inc., Brookings, SD, USA). The technicians conducting this assay were blinded to the source of material.

### TUNEL assay

To assess sperm DNA fragmentation, the TUNEL assay was used by the Center for Reproductive Medicine of Weill Cornell Medicine (New York, USA) as previously described [25]. For this assay, a commercially available kit was used to perform the test (in situ cell death detection kit; Roche Diagnostics, Rotkreuz, Switzerland). Briefly, slides were smeared with 5 μL of the semen sample from before and after each selection method and left to dry overnight. The slides were then placed in 4% paraformaldehyde for 1 h for fixation and subsequently washed with PBS and left to dry overnight once more. They were subsequently immersed for 2 min at 4 °C in a permeabilization solution comprising 0.1% Triton X-100 and 0.1% sodium citrate in PBS. The enzyme and label solutions were applied to the slides according to the kit protocol and incubated under coverslips in a humidified chamber at 37 °C for 1 h. Slides were subsequently washed in PBS, and DAPI/Antifade solution was added to visualize sperm nuclei, which were observed under a fluorescent microscope for signals indicating DNA breakage. The technicians conducting this assay were blinded to the source of the material.

### Statistics

Statistical comparisons between groups were conducted with JMP Pro 16 software (SAS Institute, Cary, NC) using one-way analysis of variance (ANOVA) with the comparison of group means assessed using the Tukey–Kramer test. For paired comparisons, the paired *t*-test was used and confirmed using a nonparametric statistic (Wilcoxon signed rank test). Differences with a probability of *p* < 0.05 were considered significant. Linear regression analyses were also performed in which case the sperm concentration data were normalized using a square root transformation as suggested by Mortimer and Lenton [26] while the TUNEL data were log_e_ transformed in order to improve the normality of their distribution. In order to account for the impact of location on the results obtained, ANCOVA was also performed using this variable as a covariate. All data are expressed as the mean value ± the standard error of the mean (SE).

## Results

### Semen quality

**Fig. 1:**
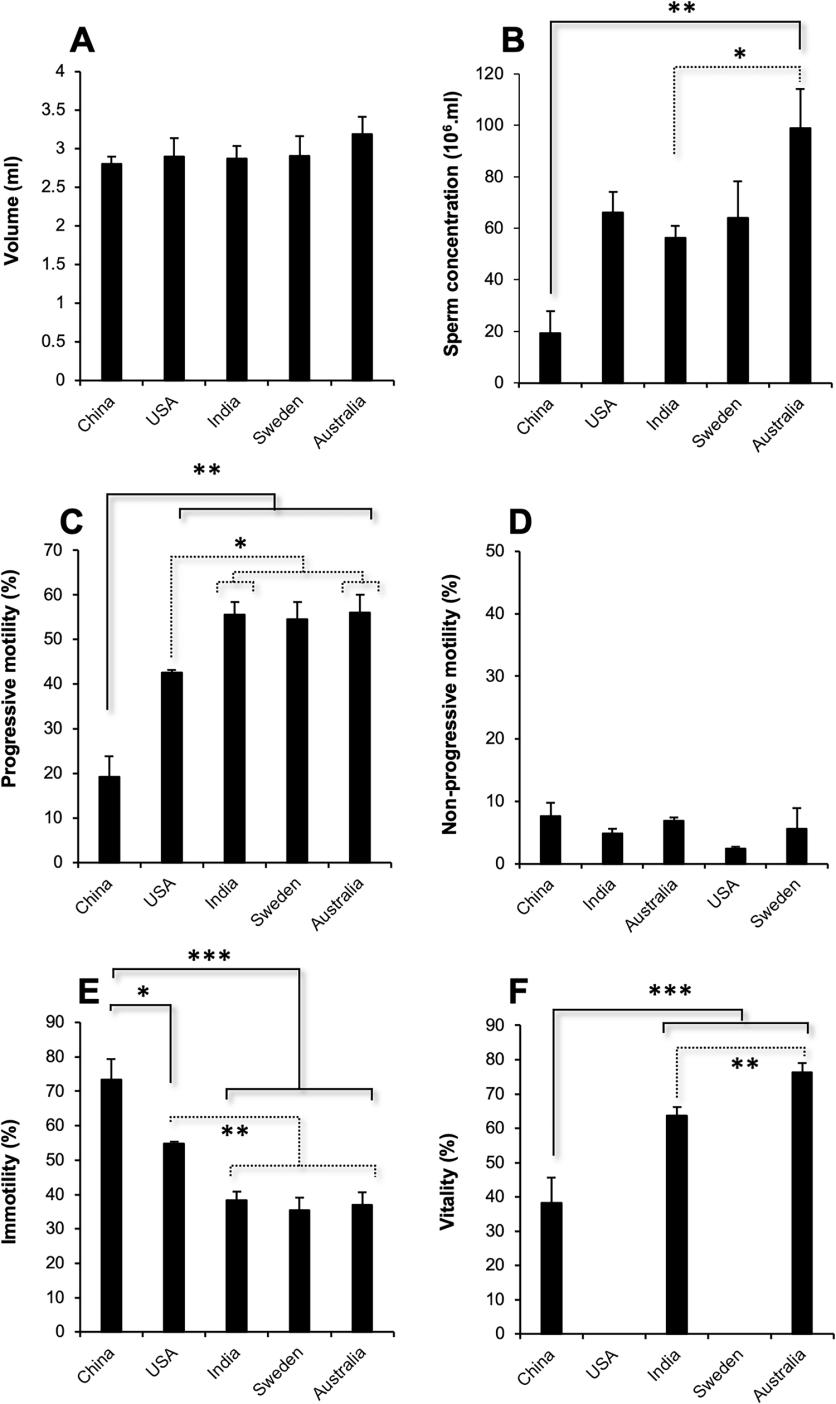
Fundamental semen profile data for the centers participating in this study. **A** Semen volume. **B** Sperm concentration. **C** Percentage progressively motile. **D** Percentage non-progressively motile. **E** Percentage immotile. **F** Percentage vital. *p-value < 0.05, ** *p*-value < 0.01, *** *p*-value < 0.001. Data represent means ± SE

Altogether, 112 semen samples were analyzed in this study. These were distributed between centers as follows: India (*n* = 38); Australia (*n* = 19); the USA (*n* = 23); Sweden (*n* = 25); and China (*n* = 7). Measurement of semen volumes revealed no significant differences between centers generating an average of 2.93 ± 0.10 mL across all samples (Fig. 1A). Sperm concentration varied significantly between centers (*p* < *0.01*) with both the Chinese and Indian datasets featuring sperm numbers that were significantly lower than those analyzed in the Australian Centre (Fig. 1B). Furthermore, progressive motility varied significantly (*p* < *0.001*) with the Chinese dataset exhibiting significantly lower progressive motility than the other centers (*p* < *0.01*) and the American cohort exhibiting lower levels of progressive motility than those observed in Australia and India (*p* < *0.05*) (Fig. 1C). In contrast, non-progressive motility was uniformly low across all centers (Fig. 1D); Levels of immotility varied between centers (*p* < *0.001*) with the Chinese and American samples exhibiting significantly higher levels of immotility than the other datasets (Fig. 1E). In those clinics where sperm vitality was routinely assessed, the Chinese samples were significantly less viable than those generated in the Indian and Australian Centres (*p* < *0.001*), while the Indian samples were less viable than those analyzed in the Australian Centre (*p* < *0.01*) (Fig. 1F).

**Fig. 2:**
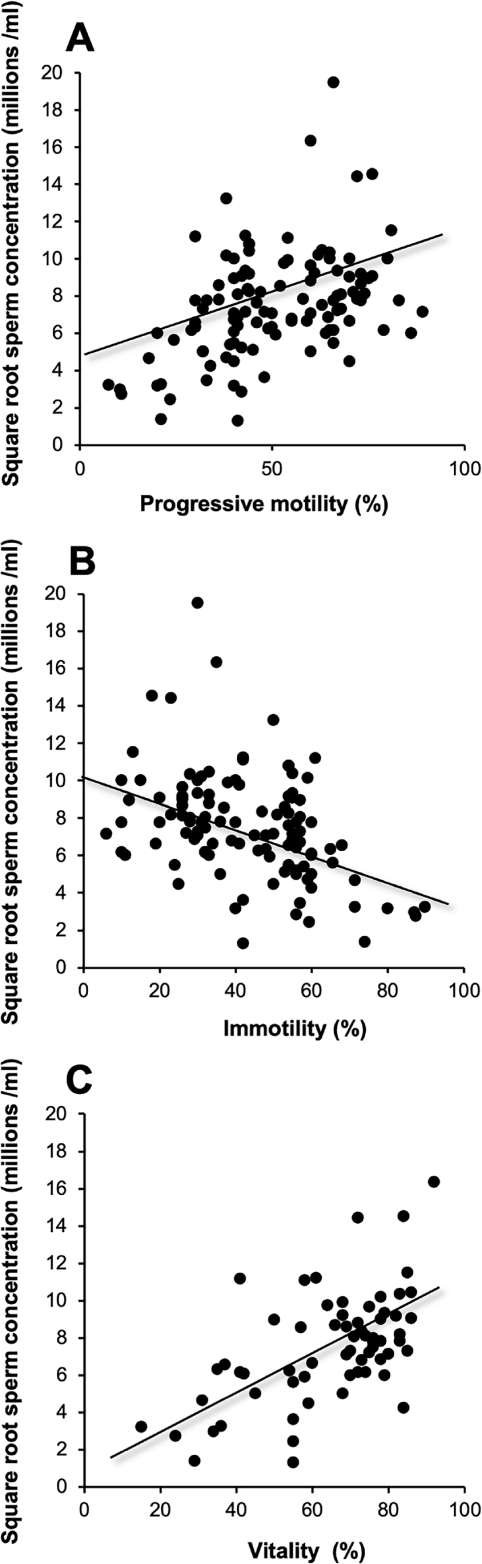
Linear correlations between sperm concentration and other parameters of semen quality across the entire data set. **A** sperm concentration and progressive motility (*R*^*2*^ = 0.19; *p*-value < 0.001). **B** Sperm concentration and immotility (*R*^2^ = 0.18; *p*-value < 0.001). **C** Sperm concentration and vitality (*R*^*2*^ = 0.33; *p*-value < 0.001). Note that the sperm concentration data have been square root transformed in order to normalize their distribution

These data therefore reveal a wide range of semen quality across all centers, with the Chinese and American clinics dealing with poorer quality samples than the other centers incorporated into this study. Importantly, across the entire dataset, significant linear correlations were observed between sperm concentration (square root transformed) and other measures of sperm quality including progressive motility (*p* < *0.001; R*^*2*^ = *0.19*), immotility (*p* < *0.001*; *R*^*2*^ = *0.18*), and vitality (*p* < *0.001; R*^*2*^ = *0.33*) (Fig. 2A–C). If ANCOVA was performed using clinic location as a covariate, then the root mean square errors rates were reduced and the strength of these associations with sperm concentration improved, generating *R*^2^ values for the regressions with progressive motility, immotility, and vitality of *R*^*2*^ = *0.30*, *0.30*, and *0.40*, respectively (*p* < *0.001*). These data suggest that despite the differences between centers in the quality of samples analyzed, across the entire dataset, there is a continuity of quality and function.

### Sperm isolation with Felix™ and DGC

**Fig. 3:**
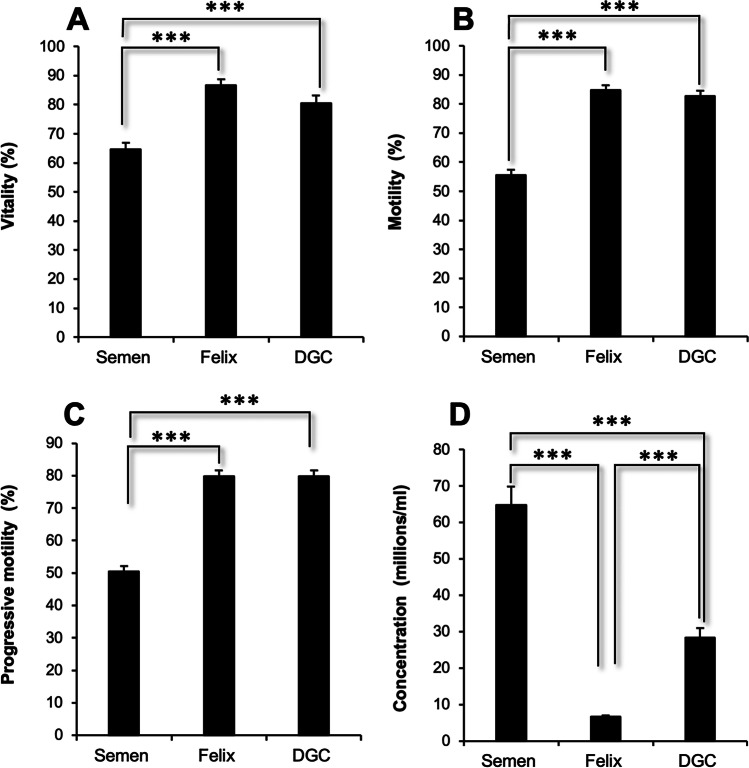
Analysis of sperm quality following isolation with the Felix™ system or DGC in comparison with the original semen sample. **A** Sperm vitality. **B** Percentage total motility (progressive + nonprogressive). **C** Percentage progressive motility. **D** Sperm concentration. *** *p*-value < 0.001. Data represent means ± SE

When these semen samples were subjected to sperm isolation using the Felix™ electrophoretic procedure or density gradient centrifugation, sperm suspensions were generated that were significantly improved relative to the corresponding unprocessed semen sample. Thus, not surprisingly, both isolation procedures succeeded in isolating spermatozoa exhibiting significantly higher levels of vitality (*p* < *0.001*), total (non-progressively motile + progressively motile) motility (*p* < *0.001*), and progressive motility (*p* < *0.001*) than the parent population (Fig. 3A–C) with no significant differences due to the isolation procedures used. However, DGC did recover a significantly larger number of spermatozoa than the electrophoretic system (*p* < *0.001*) (Fig. 3D).

### Sperm DNA damage

**Fig. 4:**
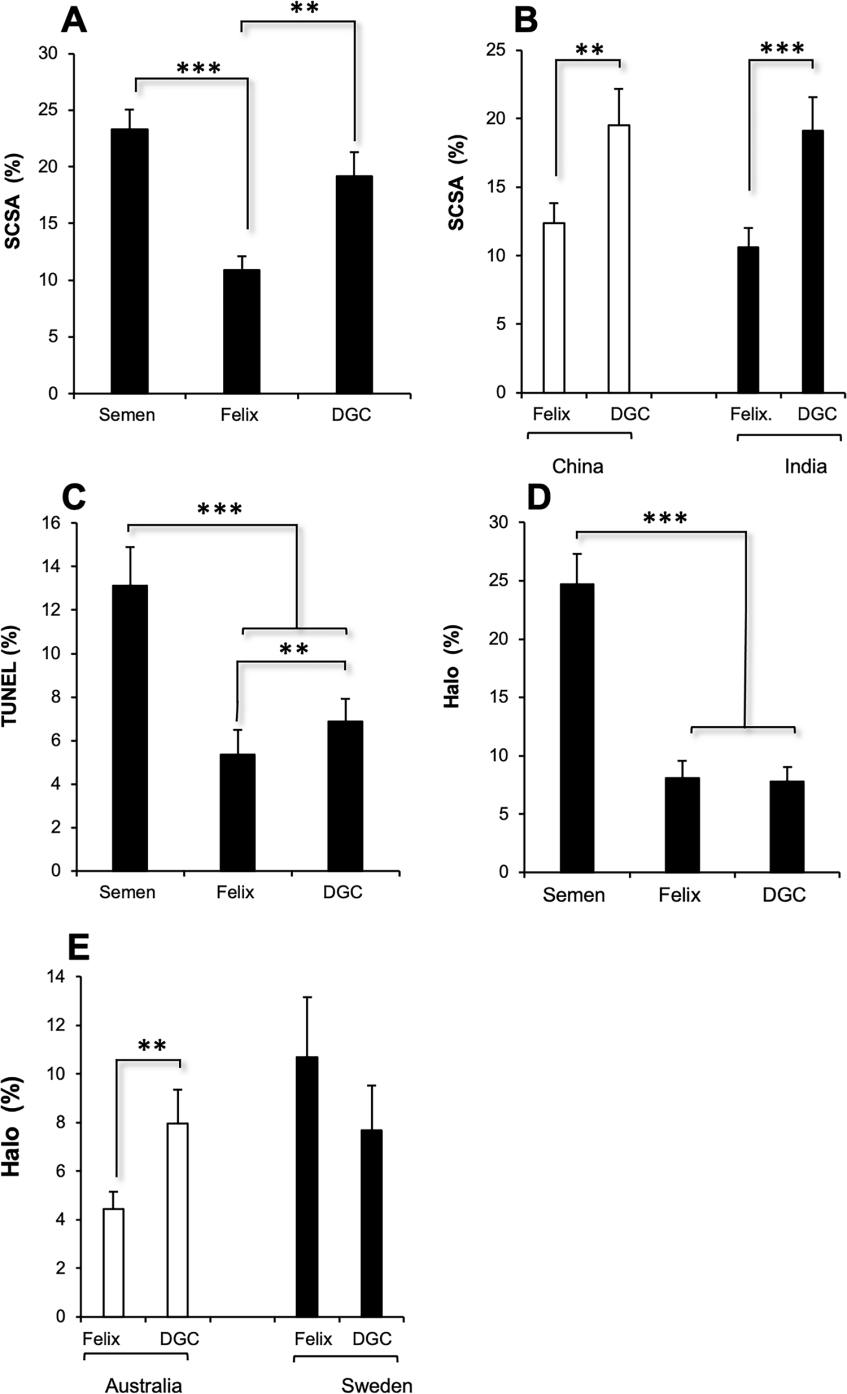
Analysis of DNA integrity in spermpopulations isolated using the Felix™ system or DGC in comparison with the original semen sample. **A** DNA damage measured by SCSA. **B** SCSA results for the 2 locations where this assay was performed showing very similar results. **C** DNA damage measured by TUNEL at the single site where this assay was performed. These data were log_e_ transformed prior to ANOVA in order to normalize the data distribution and the significance of the difference between Felix™ and DGC confirmed with both a matched pairs *t*-test and a non-parametric statistic, the Wilcoxon Sign Rank test. **D** DNA damage measured by Halo. **E** Halo outcomes for the 2 locations where this assay was performed showing discrepant results; Felix™ significantly lowered DNA damage relative to DGC in the Australian Centre not at the Swedish site where both methods of sperm isolation reduced the levels of DNA damage to a similar degree. *p-value < 0.05, ** *p*-value < 0.01, *** *p*-value < 0.001. Data represent means ± SE

In terms of DNA damage, SCSA analysis revealed a highly significant difference between the sperm isolation procedures, with the Felix™ system isolating sperm cells that had less DNA damage than the DGC isolated population (*p* < *0.001*) (Fig. 4A). Indeed, within this dataset, DNA damage in the DGC isolated population was no different from the parent population of spermatozoa in semen (Fig. 4A) Moreover, this difference between Felix™ and DGC was observed in both of the sites where SCSA was used to conduct the analysis (China and India) (Fig. 4B); confirmed using ANCOVA, which indicated no significant impact of location (*p* = *0.36*) but a significant impact of isolation procedure on the levels of DNA damage observed (*p* < *0.001*). These data were reinforced by the analysis of DNA damage using the TUNEL assay at the American Centre. This analysis revealed differences in the levels of DNA damage recorded in unfractionated semen and the sperm populations isolated by DGC and Felix™ (*p* < *0.001*) (Fig. 4C), while comparison of the DGC and Felix™ populations revealed lower levels of DNA fragmentation in the latter, that was statistically significant (*p* < *0.01*) and confirmed with a non-parametric statistic, the Wilcoxon Signed rank test (*p* < *0.01*). The Halo analysis again suggested that both DGC and Felix™ were capable of isolating subpopulations of spermatozoa exhibiting low levels of DNA damage (*p* < *0.001*) (Fig. 4D). However, in this case, there was a difference between locations as indicated by ANCOVA using location as a covariate (*p* < 0.001), with the Swedish Centre recording higher Halo results (18.09 ± 2.0; *n* = 75) than the Australian site (7.16 ± 0.700; *n* = 51) overall (*p* < *0. 001*). Moreover, there was a difference between these sites in the performance of the sperm isolation systems. At both sites, the levels of DNA damage recorded with the Halo assay were equivalent (~ 8%). However, at the Australian site, the Felix™ system generated significantly lower DNA damage values than DGC (*p* < *0.01*) while at the Swedish site, this was not the case.

## Discussion

This is the first report to examine the quality of spermatozoa recovered with the Felix™ electrophoretic system and to compare the results with a more traditional method of sperm separation, density gradient centrifugation. The Felix™ device differs significantly from previous electrophoretic sperm isolation devices reported in the literature [21–23] in that it features proprietary restriction membranes separating the electrode and sample chambers, possesses carbon rather than platinum electrodes, and has a static rather than a circulating buffer system. One of the major advantages offered by this system relative to other methods of sperm separation such as DGC is its time efficiency. From the loading of the sample chamber to the recovery of the spermatozoa takes exactly 6 min. Moreover, the Felix™ process is standardized. In contrast, there is a marked lack of standardized sperm isolation protocols when DGC is employed, as evidenced by the data provided in Table 1. The information provided by the centers participating in this study revealed that, in addition to the time taken to prepare the gradients, the most commonly employed DGC protocols employed an initial centrifugation period of 20 min followed by a further 10 min centrifugation to separate the spermatozoa from the density gradient medium and resuspend the cells in a medium suitable for assisted conception. Altogether, the separation of spermatozoa by DGC will typically take at least 40 min and will involve the exposure of spermatozoa to the physical shearing forces associated with centrifugation as well as potential attack by transition metals in the separation medium that may compromise the functionality and genetic integrity of these cells [20]. By contrast, the Felix™ system is a standardized procedure that takes a matter of minutes to complete and does not involve exposure of the spermatozoa to any extraneous chemicals other than those found in conventional IVF media.

It is well established that separating spermatozoa on the basis of their density isolates subpopulations of cells expressing high levels of progressive motility [3] This was confirmed in the present study where the efficient selection of progressively motile, vital cells following DGC was evident at all of the centers involved in this trial. Similarly, the selection of spermatozoa on the basis of a different set of principles (size, charge, and intrinsic motility) with the Felix™ device generated sperm populations exhibiting the same enhancement of movement and vitality (Fig. 3A–C). This suggests that properties such as net charge, motility, and density must be correlated possibly because all of these attributes (sialylation of proteins in the case of charge [27] and retention of excess residual cytoplasm [28] in the case of density) reflect the quality of the underlying processes of spermatogenesis and epididymal maturation. However, a major difference between the sperm isolation techniques could be found in the number of spermatozoa recovered, with DGC recovering significantly more cells (28.3 ± 2.67 million/mL) than the Felix™ system (6.74 ± 0.76 million/mL) (Fig. 3D).

The increased number of cells recovered with DGC was achieved at the expense of sperm quality as revealed by the analysis of DNA damage. In this study, 3 different techniques were used to measure the status of nuclear DNA in the spermatozoa. For two of the techniques (Halo and SCSA), the samples were immediately snap frozen in liquid nitrogen at − 196 °C, as recommended by the pioneers responsible for developing these tests [29, 30]. The SCSA system was employed in 2 centers and essentially reflects the proportion of cells possessing acid labile sites that will yield single stranded DNA on exposure to extremely low pH. In other words, it is not just measuring pre-existing strand breaks but also those breaks that are created following acid denaturation [29]. The sperm chromatin dispersal or Halo assay is similar in the sense that it also involves the lysis of spermatozoa in an acid solution. However, the end point is different. In normal cells possessing intact DNA, the release of torsional stress at the moment of cell lysis causes DNA loops to spring out of the nucleus creating halos of chromatin that can be easily visualized with DNA-sensitive dyes. Conversely, sperm with fragmented DNA do not produce halos or produce halos that are very small. The availability of commercial kits to perform this assay helps to achieve a high level of standardization, while the concordance between the Halo and SCSA assays is extremely high [31]. The third assay employed in this study, the TUNEL assay, detects DNA breakage by labeling free 3ʹ-hydroxyl termini at strand breaks [32]. It is therefore limited to measuring pre-existing strand breaks but, nevertheless, the results correlate strongly with both the SCSA and Halo assays [33]. Of the 5 centers participating in this study, 4 detected a significant improvement in the levels of DNA integrity in the Felix™-isolated cells in comparison with the DGC using all 3 techniques of assessing DNA damage. In the 5th center (Sweden), both DGC- and Felix™-isolated spermatozoa possessed lower levels of DNA damage than those observed in the original semen sample. However, no significant difference between these isolation techniques was detected. The reason why this center was an outlier in this analysis is difficult to determine. Although this was the only center where the patients had the option of producing semen samples in the comfort of their own homes, the DGC results were very similar to the other center employing the Halo assay, suggesting that the mode of semen transport was not a major issue, even though storage of human semen samples at ambient temperature is known to increase DNA damage with the Halo assay [30]. Capacitation is known to affect the negative charge associated with mammalian spermatozoa. However, there is no reason why this process should have been initiated prematurely in the Swedish Centre, given the abundant presence of decapacitation factors in human semen [34]. Factors such as ambient temperature and medium pH will also affect the behavior of spermatozoa in an electric field [35, 36] and may have been involved in generating these discrepant results with the Halo assay. Future studies will have to examine whether variation in such factors significantly influences the performance of the electrophoretic sperm isolation system, particularly with respect to DNA quality.

A limitation of this study is that it did not involve a detailed comparison with other forms of sperm isolation. Thus, while DGC is one of the most commonly used methods of sperm preparation in an assisted reproductive context, it will be important to compare Felix™ with the other frequently employed procedures, such as swim-up, as well as with the new generation of microfluidic devices that are currently appearing on the market. Such comparisons are the intended focus of future studies. From a clinical perspective, the improved DNA quality achieved with the Felix™ will be particularly useful in the context of IVF and particularly ICSI where the quality of the sperm DNA may have an important impact on the health and well-being of the offspring by influencing the latter’s mutational load (Aitken, 2022). However, this increase in quality is achieved at the expense of sperm number and this may be a problem for procedures such as intra-uterine insemination where sperm count, concentration, and total number of motile spermatozoa are critical variables in defining the success of such treatment [37].

## Conclusion

In summary, this study has compared the ability of DGC protocols used in clinical practice with the ability of the Felix™ system to isolate high-quality spermatozoa for assisted conception purposes. The project comprised an international collaboration involving 5 clinically active centers in 5 different countries. There were significant differences between clinics in the semen samples subjected to analysis. As a result, the overall dataset was representative of a wide range of semen qualities that varied in a continuous manner across all centers generating significant linear correlations between sperm concentration, movement, and vitality. Across this richly varied range of semen quality, DGC and Felix™ were consistently able to isolate sperm populations exhibiting significantly elevated levels of total motility, progressive motility, and vitality in comparison to the original semen samples. DGC generated higher levels of sperm recovery than Felix™ but even with the latter, sufficient spermatozoa were recovered for both IVF and ICSI therapy. A significant difference between DGC and Felix™ was also apparent in the levels of DNA integrity observed in the isolated sperm populations. In the context of ART, particularly ICSI, the enhanced DNA quality observed in the electrophoretically isolated cells, plus the speed with which the sperm isolation is achieved, represent significant advantages from a clinical perspective

## Data Availability

The data in the current study are available from the corresponding author on reasonable request.
